# Modelling the emergence of synchrony from decentralized rhythmic interactions in animal communication

**DOI:** 10.1098/rspb.2023.0876

**Published:** 2023-07-19

**Authors:** Yannick Jadoul, Andrea Ravignani

**Affiliations:** ^1^ Comparative Bioacoustics Research Group, Max Planck Institute for Psycholinguistics, Wundtlaan 1, Nijmegen 6525 XD, The Netherlands; ^2^ Center for Music in the Brain, Department of Clinical Medicine, Aarhus University, 8000 Aarhus, Denmark; ^3^ Department of Human Neurosciences, Sapienza University of Rome, 00161 Rome, Italy

## Abstract

To communicate, an animal's strategic timing of rhythmic signals is crucial. Evolutionary, game-theoretical, and dynamical systems models can shed light on the interaction between individuals and the associated costs and benefits of signalling at a specific time. Mathematical models that study rhythmic interactions from a strategic or evolutionary perspective are rare in animal communication research. But new inspiration may come from a recent game theory model of how group synchrony emerges from local interactions of oscillatory neurons. In the study, the authors analyse when the benefit of joint synchronization outweighs the cost of individual neurons sending electrical signals to each other. They postulate there is a benefit for pairs of neurons to fire together and a cost for a neuron to communicate. The resulting model delivers a variant of a classical dynamical system, the Kuramoto model. Here, we present an accessible overview of the Kuramoto model and evolutionary game theory, and of the 'oscillatory neurons' model. We interpret the model's results and discuss the advantages and limitations of using this particular model in the context of animal rhythmic communication. Finally, we sketch potential future directions and discuss the need to further combine evolutionary dynamics, game theory and rhythmic processes in animal communication studies.

**1. THE KURAMOTO MODEL AND (EVOLUTIONARY) GAME THEORY**

How do animals strategically time their rhythmic signals? What are the costs and benefits of signalling at a specific time? When is it evolutionary advantageous to synchronize with others? How does rhythmic interaction evolve in communication? Evolutionary, game-theoretical and dynamical systems (oscillatory) models may provide an answer to these questions. Mathematical frameworks combining the periodic nature of rhythmic interactions with the strategic or evolutionary angle of game theory are rare in animal communication research. But a recent mathematical model, developed to model something quite different, may change this scenario. In their paper, Tripp *et al*. [1] use game theory to model how group synchrony emerges from local interactions of oscillatory neurons. They show that, under a range of conditions, the benefit of joint synchronization outweighs the cost of individual neurons sending electrical signals to each other. The authors postulate there is a benefit for pairs of neurons to fire together and a cost for a neuron to communicate; their model delivers a variant of a dynamical systems classic, the Kuramoto model. How is this related to animal communication? Below we give a simple overview of the Kuramoto model and evolutionary game theory (EGT). Similarly, we summarize Tripp *et al*.'s model avoiding mathematical formalisms [1]. We then proceed to describe this model in terms of animal communication, translating parameters and assumptions from populations of neurons to interacting individuals. We discuss the advantages and limitations of using this particular model to capture animal rhythmic communication. We conclude by sketching potential future directions linking evolutionary dynamics, game theory and rhythmic processes.

The *Kuramoto model* describes the synchronization process between two or more coupled oscillators with their own initial frequency and phase [2,3]. Among other conditions, if the coupling strength is high enough, every oscillator's phase offset gets continuously nudged closer to each of its coupled oscillators' phases, and this complex system gradually ends up converging to a single shared frequency. The Kuramoto model shares a key feature with EGT models: a complex system of interacting individuals results in non-trivial emergent behaviour of the whole population. The typical example of a physical system following Kuramoto dynamics is a set of multiple out-of-sync metronomes that are physically coupled by being placed on a moving base. Each metronome's changing momentum contributes to the movement of the base, so all of the metronomes influence each other. This mutual influence results in the metronomes synchronizing over time until they are all in phase (e.g. https://youtu.be/Aaxw4zbULMs). The Kuramoto model has been used in physiology, neuroscience, biology and psychology. This fairly simple model can explain equally well synchronization among heart pacemaker cells, neurons, chirping crickets and clapping humans [2,3].

*Game theory* is a mathematical framework to analyse the strategic interaction between 2 or more individuals presented with a range of choices. In a typical game-theoretical situation (game), an individual (player) is offered a choice between several options (actions); crucially, the result of picking an action (payoff) depends on the actions chosen by all other players. Each agent thus needs a rule to pick which action to play in each situation (strategy). Determining the optimal strategy for a given game is not always intuitive nor straightforward, and game theory successfully captures the ‘if they do this, then I do that, but then they would do this, so I would do that, and … ’ recursive nature of certain inter-individual interactions. In short, a game is a static model of the possible situations arising from the interaction between different players, assigning a single numeric value to a player's utility of each outcome. Given such a game, the core aim of a game-theoretical analysis is then to predict and explain the—potentially complex—dynamics of interacting players and their strategies.

The snowdrift game (also known as the chicken game) is a well-known example of a strategic setting studied in game theory: as a simple example, imagine two neighbours needing to clean the snow from their shared part of the street. Both neighbours need to decide whether to get up early and clean, without being able to communicate to each other beforehand. If both of them decide to help, the effort will be shared and the street will be freed up (both receiving a neutral payoff of 0). If neither of them cleans it up, the snow stays, making them both late for work (a strongly negative payoff of −10 for both). However, if only one of the neighbours gets up to do the job, the work is not shared (a small negative payoff of −1), while the other neighbour manages to sleep longer and still manages to get to work on time (a small positive payoff of +1).

Game theory captures the inherent conflict in the above snowdrift game: whereas the most fair outcome would be to share the work, each player is tempted not to help and to get a better payoff in this way, so both players choosing to help is not a completely stable situation (i.e. not a ‘Nash equilibrium’). Conversely, if both players decide not to help, the outcome for both is way worse than just cleaning the street alone. If both players try to maximize their expected payoff and expect the other player to do so as well (rational players), game-theoretical analysis shows that they should clean the street 90% of the time (chosen at random). Importantly, this analysis includes the recursive nature of a player's reasoning: if one neighbour knows the other would always choose to clean the snow, there would never be a reason to help. However, knowing that the neighbour would also know that (etc. all the recursive way down) results in a perhaps unintuitive 90%–10% mixed strategy equilibrium. The analysis results of this simple game with only two players and two actions demonstrate the necessity of approaching such complex interactions within a solid theoretical framework.

EGT builds further upon these games; it models what happens when individuals' strategies may change due to repeated interactions within a population. Turning the accumulated payoff of an individual across the played games into an evolutionary fitness of the player and strategy, EGT models the evolutionary dynamics of a population. For example, in a population of agents with ‘pure strategies’ (i.e. in the snowdrift game example above, ‘always clean’ or ‘never clean’) that uniformly randomly interact with each other (well-mixed), the never-cleaning strategy has a higher payoff and thus a higher fitness in a population of always-cleaning individuals. Over time, given some model of evolution (either through imitation, or through death and reproduction over multiple generations—as in the model by [1]), a larger and larger proportion of the population will follow the never-cleaning strategy. However, this change in the proportions of different strategies in the population inherently changes the fitness landscape and actually makes it less advantageous to not clean. Moreover, when for instance agents are placed on a grid or a complex network so that they can only play the game with their neighbours, clusters of cooperation can emerge and persist [4]. Crucially, EGT models entail a complex feedback loop between the mix of strategies in the entire population, the topology structure of individuals' interactions and the optimal strategy for a single individual. This feature highlights EGT's immense relevance to biology [5]: EGT allows the modelling of local interactions between organisms, and extrapolation and analysis of the emergent behaviour of a whole population.

**2. A GAME-THEORETICAL MODEL OF OSCILLATING NEURONS**

Tripp *et al*. [1] describe an EGT model where a finite population of oscillating neurons evolves via mutual, decentralized interactions. Each modelled neuron oscillates at the same fixed frequency with a neuron-specific phase offset, and can either communicate with all others or not. Whereas communication incurs a fixed (negative) cost, a neuron receives a (positive) reward based on its level of mutual synchronization with other neurons. Any neuron can obtain a reward, no matter its communication strategy, as long as some other (partially) synchronized neurons make the effort to communicate with it. Finally, the evolution of a population is modelled in discrete time steps in a so-called ‘Moran process’ [4,6]. The higher the payoff a neuron gets, the higher its fitness; at every step, one neuron updates its strategy, choosing a new one with a probability proportional to the relative fitness of all other neurons. The authors go on to show that this simple set-up delivers several, non-trivial evolutionary dynamics. Depending on the cost–benefit ratio for uni- and bidirectional communication and the strength of the selective evolutionary pressure, communication and synchronization between oscillators evolve differently. For example, if the reward for synchronization under unidirectional communication is close or equal to that of mutual communication, a population will, under high selective pressure, show a stable mixture between communicating and non-communicating individuals, essentially resulting in a classical snowdrift game. Intuitively, one can compare the non-communication strategy of a neuron to the cleaning-avoidance strategy from our snowdrift example above: not communicating results in a higher payoff if the other player communicates (cf. if the other player makes the effort of cleaning the snow), but runs the risk of receiving a much lower payoff if no one makes the effort to communicate (cf. if no neighbour cleans up the snow and everyone is late to work). By contrast, when the benefit of bidirectional communication is much higher, the interaction between neurons will increase overall communication and synchronization. While Kuramoto equations are not built into this model, the resulting behaviour mimics Kuramoto-like dynamics.

The approach and results from Tripp *et al*. [1] could be translated into an animal communication framework as follows. There are several signalling individuals in a population. Each signaller produces sounds rhythmically (specifically, isochronously [7]), at most once per time period, at a given frequency; for simplicity these frequencies are assumed to be all the same. Just like in Tripp *et al*. ’s model, each individual has a strategy composed by a communicative action and a phase. The communicative action is to produce versus not to produce a signal during that time period. The phase is the time delay from the beginning of the period to the signal emitted. If an individual takes the action of producing a signal in a time period, it gains a benefit from synchronizing that is proportional to the degree of synchronization: the closer the phase of an individual to its neighbour, the higher its benefit [8]. However, producing a signal also entails a cost, for instance energy expenditure [9]. The combination of one's own strategy, relative cost and others' strategies delivers a payoff; a higher payoff confers higher fitness to the individual. Payoffs are only (partially) attributed if at least one individual produces a signal. At the end of a time period, one individual chosen at random will imitate another strategy present in the population, proportionally to the fitness of each strategy in the population. In other words, during each time period one individual may signal (or not) with exactly the same phase delay as another high-fitness individual in the population. Mutation may also happen, meaning that one individual may choose a random communicative action and a random phase.

Tripp and colleagues [1] ask a number of questions that can be interpreted in the context of animal communication. Under which conditions do the benefits of synchronizing outweigh the communication costs? When will the communicative strategy spread in the population, and when will it resist invasion by non-communicative individuals? When will the whole population converge towards synchrony, and when is a communicating population stable and robust to the introduction of a non-communicating individual? The answers to these questions are connected, under some simplifying assumptions (for instance, a large enough population). First, for low rates of random mutation, after multiple time periods, all individuals will start producing sounds if the benefit of mutual communication is more than double the cost to communicate. Second, under a sufficiently strong selection pressure, a non-communicative population will evolve communication if the payoff of unilateral communication outweighs its cost. Crucially, synchrony among signallers emerges under several conditions as a result of local, individual decisions, similarly to what the Kuramoto model predicts. On a more general level, the authors show the potential of this simple model to map a complex, non-trivial evolutionary dynamic. As such, a general takeaway for animal communication is the continued importance of mathematical and computational modelling of observed phenomena and proposed explanations; without doing so, it might be impossible to get sufficient insight into the evolution of communicative traits.

**3. GAME THEORY AND RHYTHMIC INTERACTIONS IN ANIMAL COMMUNICATION**

Why are these models and results relevant to understanding animal communication? Rhythm in animal communication is often interactive by definition; studying individual rhythmicity without taking the interaction with others into account risks missing out on a crucial aspect of the signalling behaviour. Interactions between 2 or more simple agents get quickly caught in feedback loops and generate complex emergent behaviour, so the ‘outcome’ of such interactions (and models) may become unpredictable [10]. And here comes the importance of game theory and evolutionary dynamics: a key property of EGT models is that they are decentralized and describe ‘local’ interactions. EGT extrapolates local interactions between simple agents to a whole population. EGT techniques also embody a feature that characterizes communication: communication is an inherently decentralized phenomenon. This makes EGT not just relevant but also crucial to the study of rhythmic interactions.

A combined rhythmic and EGT approach is needed to model interactive animal communication. Modelling work in animal communicative rhythms until now has built upon: (i) the Kuramoto equation, dynamical systems and other approaches from physics, which already make assumptions of rhythmicity and hence cannot capture its emergence via strategies as in EGT; (ii) individuals modelled as neurally inspired oscillators who interact in two-strategies games [10]; and (iii) static one-shot games, which unfortunately do not easily capture rhythmic dynamics. Instead, combining rhythm concepts with repeated evolutionary games seems a good test bench to witness the emergence of synchrony and other group dynamics.

Some aspects of the current model [1] fit well with animal communication scenarios, such as (i) the oscillatory nature of the signals; (ii) the fact that the rhythmic sequences would keep ‘oscillating in the head of the signaller’ even if a vocalization is not produced at a specific time period; (iii) the all or none communication strategy, which was adopted by the authors for simplicity; (this last simplification may fit animal communication even better than their original modelling scenario; in fact, if an animal is vocalizing in a group, the sound may reach all members of the group); and (iv) the constraint on the payoff function, where unilateral communication is no better than bilateral one. In addition, one feature unites the frameworks by Tripp and colleagues and most animal signalling: coordination without cooperation. In both cases, *cooperation is not needed* to achieve temporal, rhythmic coordination (be it synchrony or turn-taking), which instead emerges in a decentralized way [10,11]. True, the payoff function incentivizes overlap, but there is no other backchannel to establish coordination via cooperation. Rather, the coordination originates through the selfish choices of individual rational players in this game; this coordination happens because the benefits of even a moderate amount of communicative effort quickly outweigh its cost. This observation leads to a more philosophical point indirectly supported by Tripp *et al*. [1]: for a game that delivers decentralized, emergent synchronous behaviour, it is neither necessary nor scientifically parsimonious to invoke cooperation [11].

There are aspects of the current model [1] that could be adjusted to better fit animal communication scenarios, namely: (i) implementing some physiological constraints: e.g. if, at time period *t,* an individual vocalizes with a phase delay of 359 degrees, it is unlikely that the individual will be capable of vocalizing at two degrees during period *t* + 1 as the time between the two vocalizations may be physiologically too short; (ii) allowing different individuals to have different oscillatory frequencies and (iii) adding neurobiological relevance: a downside of using this EGT model for animal communication is that, mechanistically, it may be far from the neurobiological processes in animal behaviour. Finally, the more empirically minded reader may criticize the scope of the simplifying assumptions made by Tripp *et al*.'s [1]. For example, all communication is binary (either full communication or none) and a neuron either communicates with all others or not at all. As in all modelling, there is a tradeoff between realism and details versus the need to solve, analyse and interpret the models. The simplifying assumptions made by Tripp *et al*. allow the evolutionary model to be solved analytically with mathematical techniques, whereas a more complicated model would need to rely on computational simulations to investigate its evolutionary dynamics [12]. Ultimately, deciding the exact complexity of a model strongly depends on the goal of that model within the larger scientific context. Nevertheless, whatever the level of abstraction in an evolutionary model, EGT provides a framework and tools to guide future efforts into understanding communicative rhythm dynamics (as pioneering work already suggested more than two decades ago [13]). Some simplifying assumptions acknowledged by Tripp *et al*. could be explored in future research. For example, important to the context of animal communication might be to model the social network of the population's interactions. How strong is the network effect on the evolutionary process, and what happens to the typical networks we see in nature?

Finally, there is a specific animal communication framework in need of modelling that still cannot be captured by the current framework: the origins of synchrony in our hominin ancestors. Why do we have a peculiar propensity to synchronize our behaviour, be it vocal or not, with conspecifics? Based on comparative evidence from chimpanzees and bonobos (genus *Pan*), Merker *et al*. [14] try to solve the apparent paradox of our hominin ancestors displaying strong synchronization capacities at some point in evolution, with chimpanzees and bonobos lacking them. Chimpanzees and bonobos, whose rhythmic synchronization capacities are strikingly less developed than in humans, may have an ecological context that does not provide the right evolutionary incentives to overcome the communicative and cognitive cost associated with synchronization. Future EGT work seems appropriate to, and should try to, explain this conundrum [15].

## Data Availability

This article has no additional data.
